# CO_2_ Photoreduction Improvement by Carbon Nitride Utilizing the Synergism of Na Ion and Cyano Defects

**DOI:** 10.1002/open.202400431

**Published:** 2025-02-14

**Authors:** Weize Li, Zhizhong Hu, Lingyong Song, Yangbo Lv, Jincang Liu, Changtong Lu, Chunping Xu

**Affiliations:** ^1^ Technical Center of China Tobacco Guangxi Industrial Co. Ltd Nanning 530001 China; ^2^ Technical Center of China Tobacco Henan Industrial Co. Ltd. Zhengzhou 450016 China; ^3^ College of Tobacco Science and Engineering Zhengzhou University of Light Industry Zhengzhou 450002 China

**Keywords:** Cyano defect, g-C_3_N_4_, Melton salt, Na ion doping, Photocatalytic CO_2_ reduction

## Abstract

Conversion of CO_2_ to high value products was considered as a focused issue towards carbon neutrality. Photocatalysis held the potential to realize the target, and graphitic carbon nitride (g‐C_3_N_4_) was a competitive candidate. The photocatalytic efficiency of g‐C_3_N_4_ limited by the weak adsorption of CO_2_ and easy recombination of charge carriers. Herein, Na ion and cyano defects was induced into g‐C_3_N_4_ simultaneously using NaHSO_3_ as alkali molten salt to work out these obstacles. The optimized photocatalyst 3 Na‐CN (the weight of NaHSO_3_ was 1.5 g) exhibited the highest CO yield (21.5 μmol g^−1^ h^−1^), which was 5 times than that of pristine g‐C_3_N_4_ (4.29 μmol g^−1^ h^−1^). By means of experiments and characterization, 3 Na‐CN displayed better performance in both light utilization and charge separation, which was reflected by the improved photocurrent response, decreased electrochemical impedance, markedly diminished fluorescence intensity, and shortened fluorescence lifetime. This result could be ascribed to the facilitation of electron‐hole separation induced by cyano defects, as well as the enhancement in CO_2_ adsorption and activation mediated by the Na ion. This work offers a new perspective on dual modulation of graphitic carbon nitride and paves the way for the design of CO_2_ reduction photocatalyst.

## Graphical abstract

A carbon nitride with the synergistic effect of Na ion and cyano groups is designed for highly efficient photocatalytic CO_2_ reduction, reaching a production rate of CO with 21.5 μmol g^−1^ h^−1^. Na ion doping functions as an active site of CO_2_ reduction, and cyano defects act as electron traps, promoting photo‐induced carrier separation.

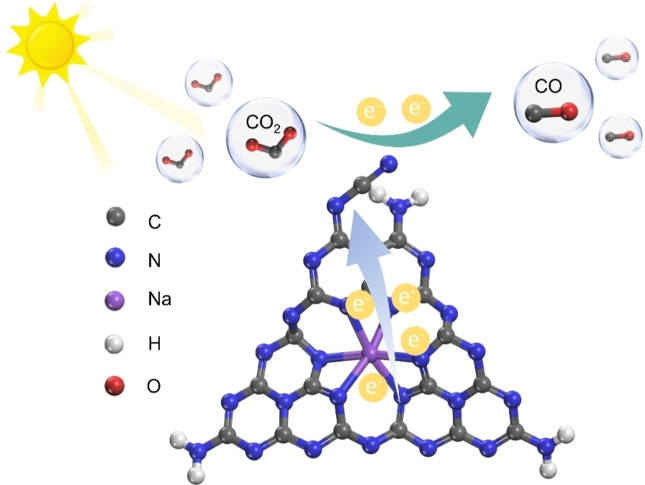

## 1 Introduction

The climate change such as greenhouse effect caused by industrial development significantly influences the carbon cycle of nature and the production of humans.^[1]^ To achieve decarbonization, carbon conversion is considered a primary technology especially converting CO_2_ into high‐value added products.^[2]^ Photocatalysis is a hopeful strategy to realize CO_2_ conversion, since its environmentally friendly superiority.^[3]^ Therefore, choosing an appropriate photocatalyst with suitable potential and easy availability is an imminent issue.^[4]^ Among those non‐metal semiconductors, graphitic carbon nitride (g‐C_3_N_4_) has been expected to stand out, owing to its excellent stability, non‐toxicity and suitable bandgap.[^5^, ^6^] Nevertheless, its practical application in photocatalytic CO_2_ reduction suffers from some restrictions, such as limited light absorption and high carriers’ recombination rate.^[7]^

In consideration of the poor efficacy of bulk photocatalysts, elements doping and defect construction have been employed as a valuable approach to boost the photoactivity of g‐C_3_N_4_. It has been reported that metal elements doping could offer extra binding energy for the catalytic system, which enhanced the light‐harvesting ability of g‐C_3_N_4_. For example, Wang et al. reported Ag doped carbon nitride (Ag‐CN) with high photocatalytic performance in bisphenol A removal.^[8]^ Ag doping reduced the band gap and enhanced the light absorption performance of g‐C_3_N_4_, expanding the absorption edge to 600 nm. According to previous research, selecting GaCl_3_ and urea as raw materials was a reliable way to obtain metal Ga‐doped g‐C_3_N_4_ (GCN).^[9]^ It could be evidently elucidated from the calculation results that the adsorption energy of H* on the Ga‐N_4_ site was positive, which means that H* is more likely to desorb from the Ga‐N_4_ site and generate H_2_. Moreover, to keep the non‐metal characterization of g‐C_3_N_4_, non‐metal elements like S,^[10]^ O,^[11]^ P^[12]^ et al were also wildly explored. Chen and co‐workers prepared S‐doped g‐C_3_N_4_ nanosheets for photocatalytic NO oxidation.^[10]^ It has been revealed that the introduction of S enhanced the light absorption, enlarged the specific area, and facilitated the separation of photo‐induced carriers.

Furthermore, S doping changed the distribution of g‐C_3_N_4_, making it easier to produce more reactive oxygen species. A report suggested that P‐doped g‐C_3_N_4_ could be synthesized through copolymerization of Guanidinium hydrochloride (GndCl) and hexachlorotriphosphazene (Cl_6_N_3_P_3_, HCCP).^[12]^ P atom replaces C atom and form chemical bonds with adjacent N atoms. The delocalization of lone pair electrons and the polarization of P−N bonds lead the generation of P^+^ center, considered as the Lewis acid sites, as well. The synergetic effect of the Lewis acid sites and the Lewis base sites facilitates the separation of electron‐hole pairs. In addition, defects engineering was unveiled as an electron or hole trapping center, which was favorable for charge separation. Chen et al synthesized K^+^ doped and N defect co‐modified carbon nitride for boosted photocatalytic H_2_ evolution. The bring‐in of K^+^ and N defects improved the light harvesting and charge separation.^[13]^

The poor charge separation, weak adsorption and activation of CO_2_ were the bottlenecks of photocatalysis. Inspired by the above research, the effect of alkali metal doping cooperating with defects engineering in CO_2_ reduction might deserve to be investigated. Alkali metal doping method was widely reported especially using Li ion and K ion, however, Na ion was an assignable candidate for its abundant storage and easy‐access. Previous work had demonstrated that K ion could play a role of electron transfer channel,^[13]^ whether Na ion also had the same property remained explore. Therefore, the aim of this work was to synchronize the introduction of sodium and cyanide defects in order to verify the effect and mechanism of sodium and cyanide on the photocatalytic CO_2_ reduction activity of g‐C_3_N_4_.

In this work, alkali molten salt NaHSO_3_ was applied to construct cyano defects and Na+ doping g‐C_3_N_4_ through secondary calcinating, and the photocatalyst was employed for CO_2_ reduction. When the mass of NaHSO_3_ was 1.5 g, the best photocatalyst (3 Na‐CN) exhibited a rate of CO yield of 21.5 μmol g^−1^ h^−1^, which is 5 times than that of bulk g‐C_3_N_4_ (CN). It was found that the synergy of Na ion and cyano defect had optimized the charge behavior, since the improvement in photochemical properties.

## Experimental Section

### Chemicals

Dicyandiamide (CP) and sodium hydrogen sulfite (CP) were purchased from Sinopharm Chemical Reagent Co. Ltd.

### Preparation of Pristine Carbon Nitride (CN)

Pristine carbon nitride (CN) was synthesized by the thermal polymerization method as generally reported. Detailly, 4 g dicyandiamide was put into a ceramic crucible with a lid and then calcinated in a muffle oven under 550 °C for 2 h in air, with a heating rate of 5 °C min^−1^. The yellow product was collected after cooling to room temperature and it was ground into fine powder.

### Preparation of g‐C_3_N_4_ with Na Ion Doping and Cyano Defects Introduction

Na ion and cyano defects co‐modified carbon nitride were fabricated through the molten salt method. 3 g CN and a certain amount of sodium hydrogen sulfite (NaHSO_3_) were mixed and grounded for 10 min. Subsequently, the mixture was calcinated under 500 °C in air for 3 h, with a heating rate of 5 °C min^−1^. The solid was gained after washing, filtering and drying. The catalysts were nominated as 1 Na‐CN, 2 Na‐CN, 3 Na‐CN and 4 Na‐CN, according to the weight of NaHSO_3_ (0.5 g, 1 g, 1.5 g and 2 g), respectively.

### Characterization of the Photocatalysts

Detailed information for photocatalysts characterizations can be found from the supporting information.

### Photocatalytic Reduction of CO_2_

Photocatalytic CO_2_ reduction was carried out using glassware (Porphyry). Detailed procedures for the photocatalytic reduction of CO_2_ can be found from the supporting information.

### In Situ DRIFTs Measurement

In situ DRIFTs measurement for photocatalytic CO_2_ reduction were performed on a FTIR spectrometer (Tensor II, Bruker, Bremen, Germany) equipped with a reaction chamber. Detailed information can be found from the supporting information.

## 2 Results and Discussion

### 2.1 Microstructure and Morphology

**Figure 1:**
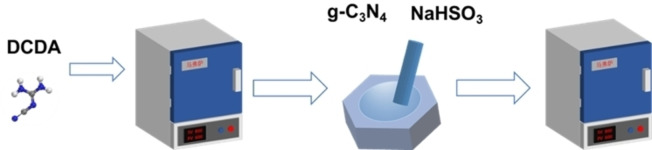
Schematic diagram of the preparation process for g‐C_3_N_4_ photocatalysts.

**Figure 2:**
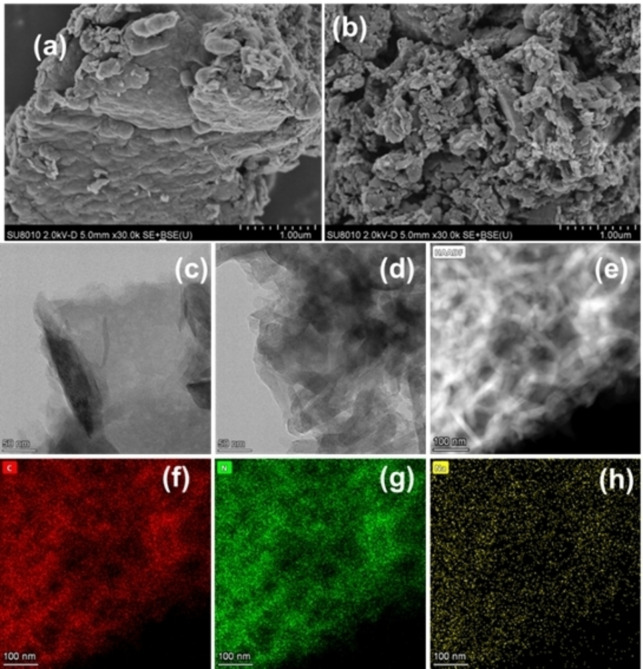
SEM & TEM images of CN (a and c) and 3 Na‐CN (b and d), HAADF (e) and the corresponding elements mapping images (f–h) of 3 Na‐CN sample.

x Na‐CN was prepared through a simultaneously calcination process (Figure 1). During the first proceeding, dicyandiamide polymerized under high temperature, while inducing cyano defects in the second procedure. The Scanning electron microscopy (SEM) images revealed a more fragmentary structure in 3 Na‐CN (Figures 2a and 2b). It was consistent with the Transmission electron microscopy (TEM) that 3 Na‐CN displayed a more uniformly stacked form, when compared to bulk CN (Figures 2c and 2d). Moreover, it could be observed that 3 Na‐CN exhibited a clear lattice fringe of 0.33 nm,^[2]^ which was related to the (002) facet (Figure S1). This phenomenon unveiled that NaHSO_3_ facilitated the re‐crystallization of CN as hard template. NaHSO_3_ decomposed at about 150 °C to form Na_2_SO_3_, then melted into molten salt at 500 °C, which provided a re‐polymerization environment for CN. Elements mapping was employed to observe the distribution of C, N and Na (Figures 2e–h). It showed that Na was homogenously doped in the photocatalysts.

The Brunauer‐Emmet‐Teller specific surface area (S_BET_) of the materials had a marked impact on CO_2_ adsorption and a profound influence on the photocatalytic CO_2_ reduction performance.^[14]^ The nitrogen adsorption‐desorption isotherms, together with the corresponding pore size distribution curves of the synthesized CN photocatalysts, were determined. As illustrated in Figure S2, the adsorption isotherms of all samples exhibited a IV type,^[15]^ and the hysteresis loops were of the H3 type in a relative pressure range of 0.8–1.0.^[16]^ All samples presented similar specific surface areas, proving that the specific surface area should not be the source of the increased activity of carbon nitride (Table S1). Therefore, the structural changes of carbon nitride were explored.

### 2.2 Phase Structure and FTIR

**Figure 3:**
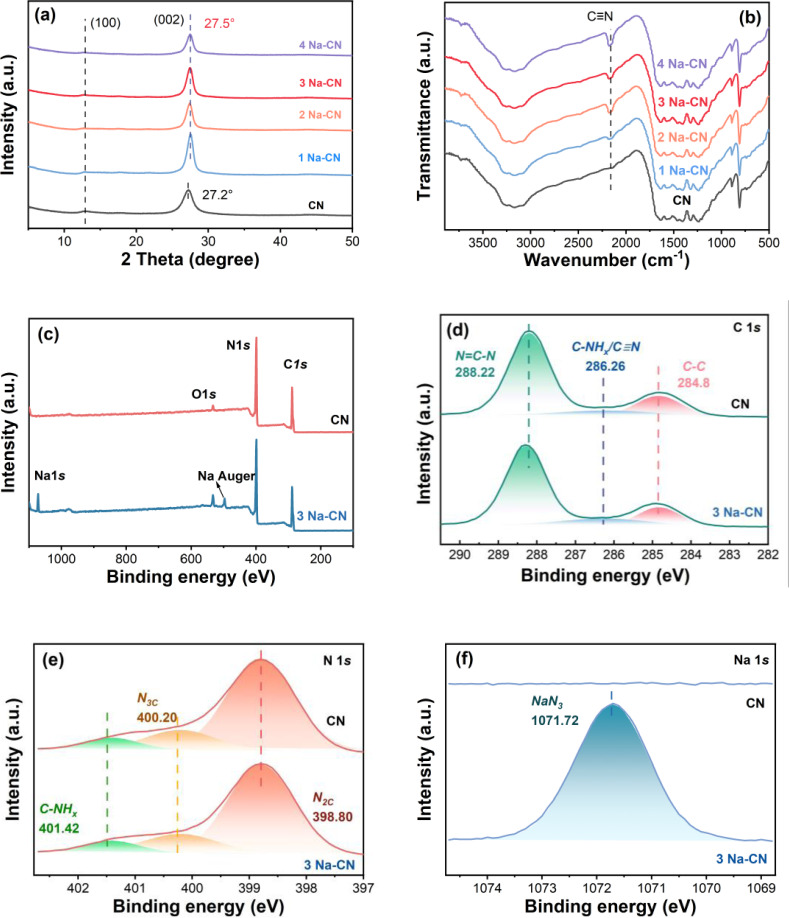
(a) XRD pattern, (b) FTIR spectra of CN and x Na‐CN. (c) XPS survey spectra of CN and 3 Na‐CN; High‐resolution XPS spectra: (d) C 1s, (e) N 1s and (f) Na 1s of CN and Na‐CN.

In order to verify the specific relationship between the structure and catalytic properties, CN and x Na‐CN were analyzed by X‐ray diffraction (XRD). Both CN and x Na‐CN showed two characteristic peaks ascribed to the (100) facet of the heptazine plane and the (002) facet of the interplane stacked structure (Figure 3a).[^17^, ^18^] It was noteworthy that the position of the (002) peak moved towards a higher angle with the increase of Na ion, indicating the decrease in the distance between layers. Meanwhile, the (002) peak became sharper after adding NaHSO_3_. These two changes could be attributed to the enhancement brought by molten salt in crystallinity. The addition of molten salt formed limited space for the growth of g‐C_3_N_4_, and g‐C_3_N_4_ aggregated more closely, that is why distinct lattice fringe could be observed.

The FT‐IR spectra of the original CN and x Na‐CN samples showed characteristic bands corresponding to heptazine ring in the range of 1200 to 1600 cm^−1^, as well as stretching vibrations of aromatic C−N bonds at approximately 810 cm^−1^ (Figure 3b).^[19]^ In contrast, the x Na‐CN sample exhibited a distinct cyano peak centered around 2160 cm^−1^, attributed to the deprotonation of the carbon‐bond amino group at the terminus of carbon nitride.^[20]^ This peak was consistently observed in all x Na‐CN samples, suggesting that the cyano defects could be retained after prolonged calcination. To investigate the reason and period of forming cyano, controlled trial was set. The controlled samples were fabricated as previous synthesis process of 3 Na‐CN, instead of the calcination temperature ranging from 100–500 °C. The gathered samples were named as y Na‐CN, where y represented temperature. Figure S3 revealed the formation condition of cyano defects. No signal of cyano defects emerged under 500 °C, indicating that cyano defects were only generated in the circumstance of molten salt.^[21]^ This is due to the fact that the synthesis of carbon nitride starts at 500 °C and above 500 °C the carbon nitride is in the free state, at which point the addition of the molten salt sodium bisulfite disrupted the structure of the originally polymerized carbon nitride, breaking the terminal C−N bond to form cyano. Furthermore, cyano could be identified in 3 Na‐CN through ^13^C NMR spectroscopy (Figure S4). In comparison to pristine CN, the NMR spectrum of 3 Na‐CN exhibited a cyano peak at δ=119 ppm and δ=180 ppm, while maintaining the fundamental heptane unit of CN.^[22]^

### 2.3 XPS Analysis

To further confirm the chemical composition, X‐ray Photoelectron Spectroscopy (XPS) was applied. C and N were both detected in CN and 3 Na‐CN, while Na was exhibited in 3 Na‐CN, which proved that Na ion was successfully doped in 3 Na‐CN (Figure 3c). In Figure 3d, CN showed three peaks at 288.22 eV, 286.26 eV and 284.8 eV which could be assigned to N=C−N in the aromatic rings of the g‐C_3_N_4_ heterocycles, C−NH_x_ (x=1, 2), and adventitious carbon,^[23]^ respectively. The area of peak at 286.26 eV enlarged because of the formation of cyano defects derived from terminal −NH_x_. Moreover, the binding energy of N=C−N moved toward a positive position could be attributed to the trap of electrons caused by cyano defects. The N 1s XPS spectra of all samples exhibited three distinct binding peaks at 398.9, 400.3, and 401.5 eV (Figure 3e). These peaks correspond to N_2C_ (C−N=C), N_3C_ (N‐3 C,), and NHx groups in the framework, respectively.^[24]^ In the Na 1s spectra, 3 Na‐CN displayed a distinct peak correlated to Na−N bonding at 1071.72 eV,^[25]^ which intuitively the coordination of Na and N in the heptazine (Figure 3f). The above results confirmed that Na ion was successfully doped into g‐C_3_N_4_ without destroying the skeleton.

### 2.4 Photocatalytic Performance on CO_2_ Reduction

**Figure 4:**
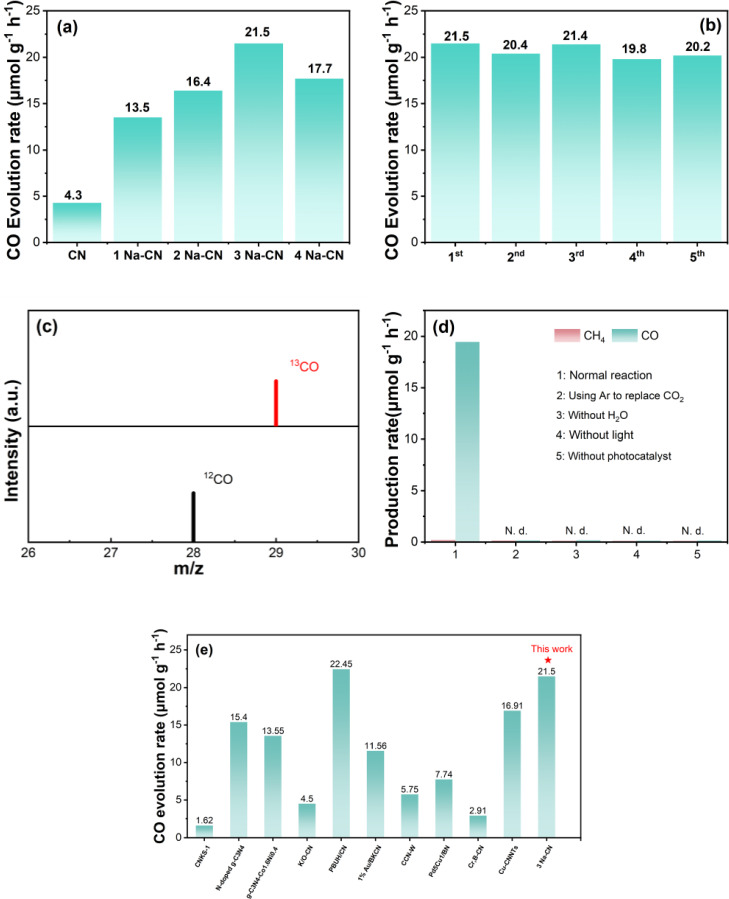
(a) Photocatalytic CO_2_ reduction rate of CN and x Na‐CN; (b) cycling test of 3 Na‐CN; (c) Mass spectrometry analysis of CO_2_ photoreduction over 3 Na‐CN with NaHCO_3_ or NaH^13^CO_3_ as carbon source; (d) product production rate over 3 Na‐CN under different reaction conditions (N.d.: Not detected); (e) comparison of CO_2_ photoreduction activity with other carbon nitride‐based photocatalysts reported in the literature (see Table S2 for details).

The photocatalytic activity of all samples was estimated in a closed quartz reactor. The optimal amount of photocatalyst was determined by conducting dosage optimization experiments. As illustrated in Figure S5, the catalyst exhibited its optimal unit activity at a dosage of 5 mg, while at dosages of 40 mg, its activity was found to be reduced. Given the operational constraints of the actual weighing process and the objective of enhancing catalyst recyclability, a dosage of 20 mg was selected as the optimal photocatalyst dosage. Figure 4a showed the photocatalytic reduction rate of CO_2_ by x Na‐CN. With the increase of NaHSO_3_, the yield of CO gradually increased. The yield of CO on 3 Na‐CN reached 21.5 μmol g^−1^ h^−1^, which was five times higher than that of bulk CN (4.3 μmol g^−1^ h^−1^). The photocatalytic conversion efficiency from CO_2_ to CO of the 3 Na‐CN sample was significantly higher compared with that of CN, and the activity began to decrease with the increase of the amount, which may be related to the excess of molten salt that makes it difficult to polymerize the catalyst during synthesis, which is in agreement with the XRD results (Figure 3a) that the (002) peak of the 4 Na‐CN becomes blunter. The photocatalytic performance of the 3 Na‐CN sample remained 90 % after 5 consecutive cycles, confirming the stability of 3 Na‐CN was excellent (Figure 4b).

Apart from the performance test, mass spectrometry was used to verify the source of CO. The condition of CO reduction test was the same as the previous test, except for the replacement of the carbon source (NaH^13^CO_3_). The results showed that ^12^CO was detected at the position of m/z=28 using NaHCO_3_, while ^13^CO was detected at the position of m/z=29 when using NaH^13^CO_3_ as a carbon source (Figure 4c). This consequence verified that CO was reduced by CO_2_ instead of conversion of other impurities. Moreover, the photocatalytic activity of 3 Na‐CN was examined by employing argon in place of CO_2_. The results demonstrated that no carbon‐containing products were produced (Figure 4d). Analogously, the absence of water, light, and catalyst resulted in no products being generated, thereby substantiating the conclusion that CO_2_ was the sole source of carbon‐containing products. A comparison of 3 Na‐CN with carbon‐nitride‐based catalysts documented in the extant literature reveals its relative superiority in terms of photocatalytic CO_2_ reduction efficiency, particularly in the absence of co‐catalysts and sacrificial agents (see Figure 4e and refer to Table S2).

### 2.5 Photochemical Properties

**Figure 5:**
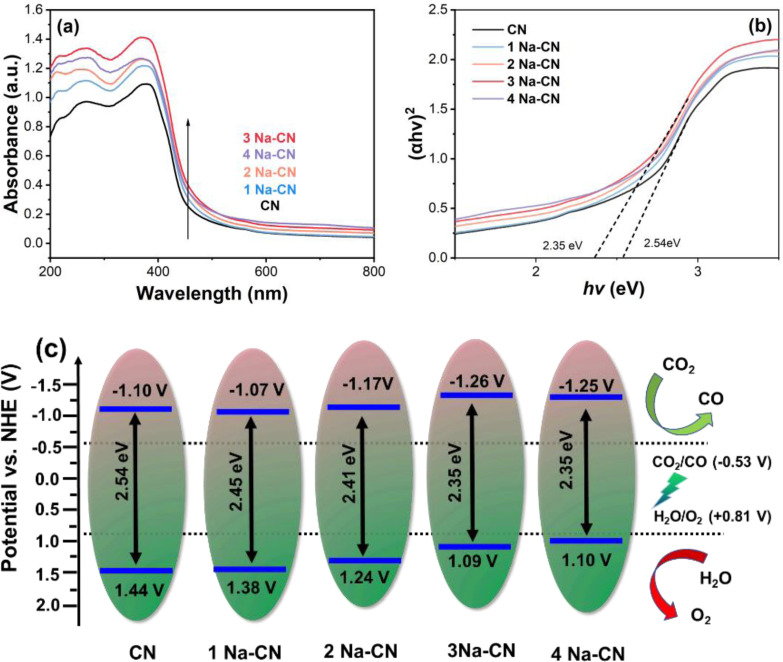
(a) UV‐vis DRS spectrum, (b) corresponding tauc plot and (c) band structure illustration of as‐prepared samples

Light‐harvesting ability was considered as one of the main factors in photocatalysis, thus, UV‐vis DRS was applied to investigate the influence brought by molten salt. From Figure 5a, it was distinct that the light absorption ability of all samples exhibited enhancement and the absorption edge displayed redshift, with the increase of NaHSO_3_. However, excess mass of molten salt broke the trend, as the 4 Na‐CN showed a decrease in light harvesting. This change probably originated from the creation of more nitrogen atom lone pairs and deformation of the planar heptazine structure due to the introduction of cyano defects, which leads to an easier excitation of n→π* orbitals.^[26]^ Combined with the Tauc diagram of the catalyst (Figure 5b) the electronic energy band configuration of the catalyst could be estimated. As the amount of NaHSO_3_ increased, their band gap decreased slightly from 2.54 to 2.35 eV. The band gaps of CN and x Na‐CN were calculated to be 2.54, 2.52, 2.48, 2.38 and 2.35 eV by tangent calculation, respectively.

The position of the conduction band (CB) was inseparable from the reduction ability of semiconductors. Mott‐Schottky plots were utilized to reveal the change in all samples. The Mott‐Schottky plots of the x NaSCN samples have positive slopes, indicating that they have typical n‐type semiconductor properties (Figure S6). The conduction band positions of 1 Na‐CN, 2 Na‐CN, 3 Na‐CN, and 4 Na‐CN were −1.32, −1.42, −1.51 and −1.44 V, respectively (vs. Ag/AgCl@pH 5.95). Combined with the band gap of the catalysts, the valence band (VB) positions of the CN and x Na‐CN samples were calculated to be 1.19, 1.13, 0.99, 0.84 and 0.85 V, respectively. The electronic energy band positions of all the samples were shown in Figure 5c. It was clear that the introduction of cyano defects would shorten the band gap of g‐C_3_N_4_, and caused a negative shift of CB. On the one hand, the narrower band gap made it easier for photoelectrons to be excited; on the other hand, the more negative conduction band potential made the photogenerated electrons more capable of reduction, both of which were favorable for the photocatalytic CO_2_ reduction reaction.^[27]^

**Figure 6:**
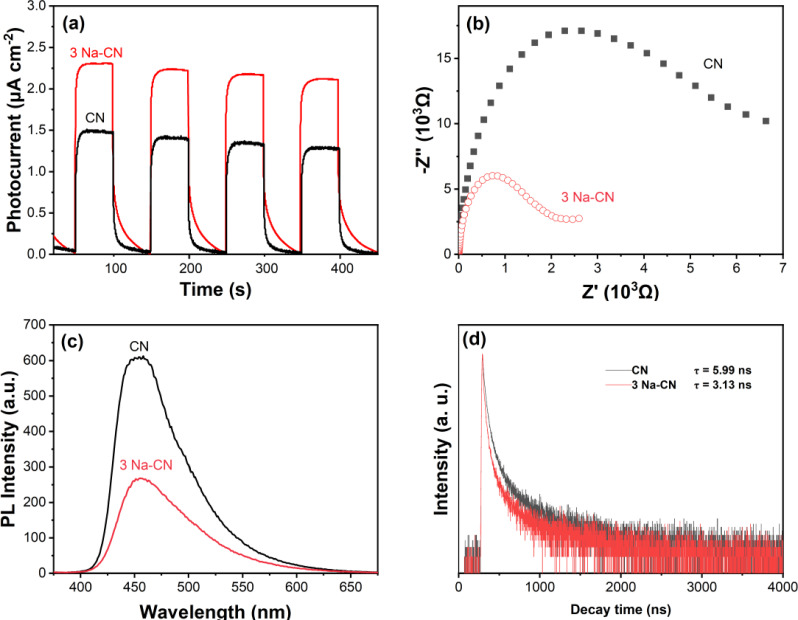
Photoeletrochemical properties of the g‐C_3_N_4_ photocatalysts: (a) curves photocurrent response, (b) spectra of electrochemical impedance, (c) spectra of steady‐state of photoluminescence and (d) spectra of time‐resolved photoluminescence.

In addition, the electron transfer performance of photocatalysts was also evaluated by fluorescence spectroscopy to assess the recombination behavior of photocarriers in as‐prepared samples. From the transient photocurrent response curves shown in Figure 6a, it could be seen that the 3 Na‐CN had a higher photocurrent response compared with pristine CN. A similar trend is obtained in the electrochemical impedance (EIS) measurements, as shown in Figure 6b. The arc radius of 3 Na‐CN is much smaller than that of pristine CN, which suggested that the carrier‐transfer resistance is significantly lower and the diffusive mobility of electrons is greatly improved.^[28]^

As shown in Figure 6c, CN showed the strongest curve intensity, indicating a relatively high recombination rate of photogenerated electrons and holes. The recombination rate in 3 Na‐CN was significantly lower, which was attributed to the fact that the Na+ doping during the synthesis process could effectively improve the crystallinity and promote charge separation. Moreover, the generated cyano could act as an electron acceptor to promote the directional movement of the photogenerated carriers. In order to obtain more information about the charge transfer kinetics, the time‐resolved fluorescence spectra of CN and 3 Na‐CN were tested (Figure 6d). Both CN and 3 Na‐CN exhibited a slower exponential decay. The fluorescence lifetime of 3 Na‐CN was 3.13 ns, which is 0.52 times that of the pristine CN (5.99 ns). The reduced fluorescence lifetimes of charge carriers would provide a longer time for the photogenerated holes and electrons to be transferred to the active sites,^[29]^ and then participate in the subsequent photocatalytic CO_2_ reduction reaction. This result further confirmed the efficient charge separation properties of 3 Na‐CN as shown in PL spectra.

### 2.6 Pathway of CO_2_ Reduction

**Figure 7:**
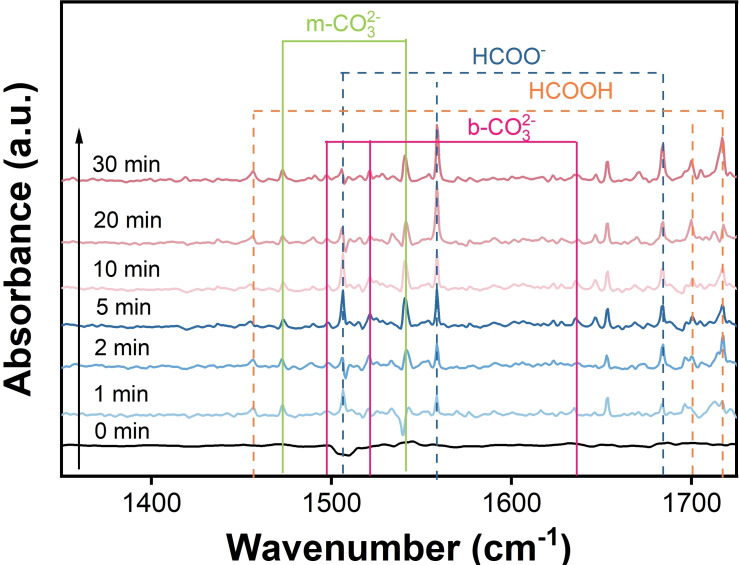
In situ DRIFTS for CO_2_ reduction over illuminated 3 Na‐CN.

Based on the outcomes of CO_2_ photoreduction tests, a potential photocatalytic CO_2_ reduction mechanism over 3 Na‐CN was initially postulated (Figure 7). No significant peak could be seen in dark, confirming that the products were derived from CO_2_. The peaks at 1472 cm^−1^ and 1540 cm^−1^ were ascribed to the monodentate carbonate (m‐CO3^2−^).^[30]^ The peaks located 1497 cm^−1^, 1521 cm^−1^ and 1635 cm^−1^ were attributed to bidentate carbonate (b‐CO3^2−^).^[31]^ These two species originated from the coordination of CO_2_ with H_2_O molecule adsorbed on the surface. The peaks lay in 1456, 1700 and 1717 cm^−1^ could be assigned to formic acid (HCOOH), while peaks at 1515, 1558 and 1684 cm^−1^ belonged to formate (HCOO^−^).^[32]^ Based on the identification of the intermediates, it can be hypothesized that formic acid and formate play a pivotal role in the reduction of CO_2_ to CO. The reaction process is illustrated in equations 1–5., 2, 3, 4

(1)

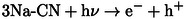



(2)

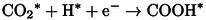



(3)

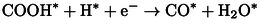

(4) $${\mathrm{CO}}^{*}\to \mathrm{CO}↑$$

Initially, CO_2_ is adsorbed on the active sites of the catalyst surface, and with the participation of electrons, CO_2_ couples with a proton, resulting in the formation of COOH* intermediates. Subsequently, the COOH* molecule couples with a hydrogen proton and is reduced by electrons to form CO* and H_2_O. Finally, CO* is desorbed from the catalyst surface, resulting in the formation of the reduction product CO.

## 3 Conclusions

In summary, Na+ doped and cyano defects induced g‐C_3_N_4_ was fabricated by the molten salt template. The experimental results demonstrated that the CO_2_ reduction reaction rate of 3 Na‐CN was (21.5 μmol g^−1^ h^−1^), which was nearly five times higher than that of bulk CN (4.3 μmol g^−1^ h^−1^). This indicated that 3 Na‐CN had excellent catalytic performance in CO_2_ reduction. The addition of NaHSO_3_ results in a decrease in the band gap of the carbon nitride and a negative shift of the conduction band position, which led to a more powerful reduction ability. 3 Na‐CN exhibited a significant enhancement of the photo‐absorption and carrier separation performances. The characterization results revealed that Na ion doping promoted the enhancement of crystallinity in CN and acted as an active site to improve the efficiency of CO_2_ conversion. In contrast, cyana defects served as electron traps, facilitating carrier separation. This dual modulation design offered a novel approach to the rational design of photocatalytic CO_2_ reduction catalysts.

## Supporting Information

Characterization of the photocatalysts, photocatalytic reduction of CO_2_, In situ DRIFTs measurement, Figure S1: TEM images of 3 Na‐CN; Table S1: BET surface area of all samples and corresponding well volume and pore size; Figure S2: (a) Nitrogen adsorption‐desorption curves and (b) pore size spectra of CN and 3 Na‐CN; Figure S3: FTIR spectra of y Na‐CN; Figure S4: ^13^C NMR of CN and 3 Na‐CN; Figure S5: Photocatalytic CO_2_ reduction with different catalyst dosages for (a) total CO production, (b) CO unit yield, and (c) kinetic profile; Table S2: CO_2_ photoreduction activities of various carbon nitride‐based photocatalysts reported in recent years; Figure S6: Mott‐Schottky plots of (a) CN, (b) 1 Na‐CN, (c) 2 Na‐CN, (d) 3 Na‐CN and (e) 4Na ‐CN.

## Author Contributions

Conceptualization, Zhizhong Hu and Chunping Xu; methodology, Changtong Lu; software, Lingyong Song; validation, Yangbo Lv; formal analysis, Weize Li; investigation, Chunping Xu; resources, Jincang Liu; data curation, Weize Li; writing–original draft preparation, Weize Li and Zhizhong Hu; writing–review and editing, Chunping Xu.; visualization, Changtong Lu; supervision, Chunping Xu; project administration, Chunping Xu; funding acquisition, Chunping Xu. All authors have read and agreed to the published version of the manuscript.

## Conflict of Interests

The authors declare no conflict of interest.

## Supporting information

As a service to our authors and readers, this journal provides supporting information supplied by the authors. Such materials are peer reviewed and may be re‐organized for online delivery, but are not copy‐edited or typeset. Technical support issues arising from supporting information (other than missing files) should be addressed to the authors.

Supporting Information

## Data Availability

Data sharing is not applicable to this article as no new data were created or analyzed in this study.
