# Supplementary material for: CO2 Photoreduction Improvement by Carbon Nitride Utilizing the Synergism of Na Ion and Cyano Defects
Source: ChemistryOpen. 2025 Feb 14;14(7):e202400431. doi: 10.1002/open.202400431 (PMC12256927; doi:10.1002/open.202400431)
Supplement: Supplementary file 1 — Supporting Information [file OPEN-14-e202400431-s001.pdf]

ChemistryOpen

Supporting Information

CO_2 Photoreduction Improvement by Carbon Nitride Utilizing the Synergism of Na Ion and Cyano Defects

Weize Li, Zhizhong Hu, Lingyong Song, Yangbo Lv, Jincang Liu, Changtong Lu,* and Chunping Xu*

Supporting Information

CO_2 Photoreduction Improvement by Carbon Nitride Utilizing the Synergism of Na ion and Cyano defects

Weize Li[1]·Zhizhong Hu[1]·Lingyong Song[1]·Yangbo Lv[1]·Jincang Liu[1]·Changtong Lu[2*]· Chunping Xu[3*]

[1] Technical Center of China Tobacco Guangxi Industrial Co. Ltd, Nanning 530001, China;

[2] Technical Center of China Tobacco Henan Industrial Co. Ltd. Zhengzhou 450016, China;

[3] College of Tobacco Science and Engineering, Zhengzhou University of Light Industry, Zhengzhou 450002, China*

Correspondence: luchangtongfly@163.com (C. L.); c.p.xu@zzuli.edu.cn (C. X.)

1. Characterizations

The obtained samples were characterized by X-ray diffraction (XRD) patterns on a Rigaku D/MAX-RB diffractometer with Cu Kα radiation (λ = 1.5418 Å). X-ray photoelectron spectroscopy (XPS) measurements were carried out on a PerkinElmer PHI 5000C system, and all XPS spectra of the as-prepared samples were calibrated by setting the C1s peak of adventitious carbon to 284.6 eV. The morphology of the BCN and CN-Cl x samples was measured by transmission electron microscopy (TEM) (FEI talos f200s). The nitrogen adsorption-desorption isotherms of the obtained samples were recorded in a nitrogen adsorption apparatus (ASAP 2020, Norcross, GA, USA). The BET surface area (SBET) of the samples was calculated by a multipoint BET method under a relative pressure range of 0.05–0.3. The corresponding pore size distribution curves were obtained by the Barret–Joyner–Halender (BJH) method. The UV-vis diffuse reflectance spectra (DRS) were collected on a UV-vis spectrophotometer (UV2550, Shimadzu, Osaka, Japan) with $BaSO_4$ as the reflectance. The steady-state photoluminescence (PL) measurement was carried out with a Fluorescence Spectrophotometer (F-7000, Hitachi, Tokyo, Japan) with an excitation wavelength of 350 nm. The electro-chemical characterizations were tested in a standard three-electrode configuration (CHI760E, Shanghai, China).

2. Photocatalytic Reduction of CO_2

Photocatalytic CO_2 reduction was carried out using glassware (Porphyry). Firstly, 20 mg of the photocatalyst was dispersed into 15 mL of water to form suspensions. Secondly, the suspensions were transferred into a petri dish (Φ = 60 mm). After ultrasonication for 10 min, the dish was moved into an electric oven for

drying overnight. The uniformly dispersed film was formed and placed in the reactor. Then, 1.2 g of sodium bicarbonate was added to the glassware. After that, the air was pumped out from the reactor. Next, 5 mL of sulfuric acid solution (2M) was injected into the reactor and reacted with sodium bicarbonate to produce carbon dioxide and water vapor in the system. After illumination under a Xenon arc lamp for 1 h, 1.0 mL of the mixed gas was syringed and injected into a gas chromatograph (GC-2014, Shimazu, Osaka, Japan) for product analysis. The obtained gas chromatograph and detailed calculation process can be found in the Supplementary Materials. When repeating the experiment, except for replacing fresh sodium bicarbonate and sulfuric acid, the other procedures were identical to the photocatalytic reaction mentioned above.

3. In Situ DRIFTs Measurement

In situ DRIFTs measurement for photocatalytic CO_2 reduction were performed on a FTIR spectrometer (Tensor II, Bruker, Bremen, Germany) equipped with a reaction chamber. Before measurement, the sample was pre-dried at 100 °C in a vacuum overnight. The dried sample was subsequently placed in the sample chamber, followed by blowing with highly pure He to replace air. After that, a mixture of CO_2 gas and H_2O vapor flowed into the sample cell for 60 min until the absorption equilibrium was achieved. The IR spectra of 3 Na-CN sample, both in the dark and under illumination, was recorded. A MCT detector was used during the measurement. The IR spectra were recorded by averaging 32 scans in the range of 600-4000 cm^{-1} with a 2 cm^{-1} resolution.

Table S1. Physical properties of the samples..

Samples	SBET (m^2 g^{-1})	PV (cm^3 g^{-1})	APS (nm)
CN	18.2	0.15	0.10
1 Na-CN	18.3	0.13	0.07
2 Na-CN	19.0	0.15	0.07
3 Na-CN	18.3	0.14	0.16
4 Na-CN	17.5	0.14	0.24

Table S2. CO_2 photoreduction activities of various carbon nitride-based photocatalysts reported in literatures.

**Photocatalyst**	**Light source**	**Reaction condition**	**CO Evolution rate (μmol g^{-1} h^{-1})**	**Reference**
CNKS-1	300 W Xe lamp	CO_2 H_2O	1.62	[1]
N-doped g-C_3N_4	300 W Xe lamp (λ<420 nm)	CO_2 H_2O	15.4	[2]
g-C_3N_4-$Co_{1.6}Ni_{0.4}$	300 W Xe lamps (λ > 400 nm)	CO_2 H_2O	13.55	[3]
K/O-CN	300 W Xe lamp	CO_2 H_2O	4.5	[4]
PBUH/CN	300 W Xe Lamp (AM 1.5)	CO_2 H_2O	22.45	[5]
1% Au/BKCN	300 W UV lamp (400 nm)	CO_2 H_2O	11.56	[6]
CCN-W	300 W Xe lamp	CO_2 H_2O	5.75	[7]
Pd_5Cu_1/BN	300 W Xe lamp	CO_2 H_2O	7.74	[8]
Cr,B-CN	300 W Xe lamp	CO_2 H_2O	2.91	[9]
Cu-CNNTs	300 W Xe lamps (λ > 420 nm)	CO_2 H_2O	16.91	[10]
3 Na-CN	300 W Xe lamp	CO_2 H_2O	21.5	This work

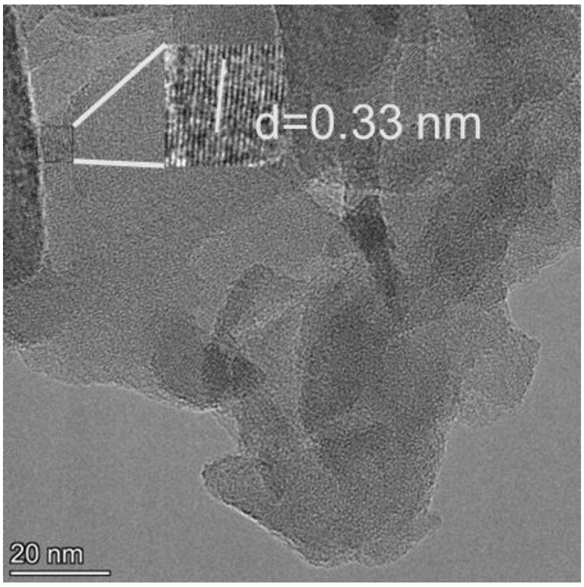

Figure S1. TEM images of 3 Na-CN.

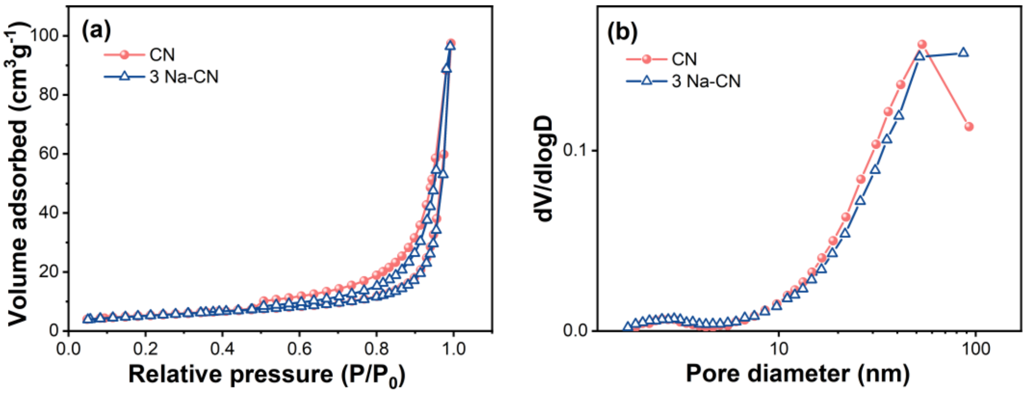

Figure S2. (a) Nitrogen adsorption-desorption curves and (b) pore size spectra of CN and 3 Na-CN.

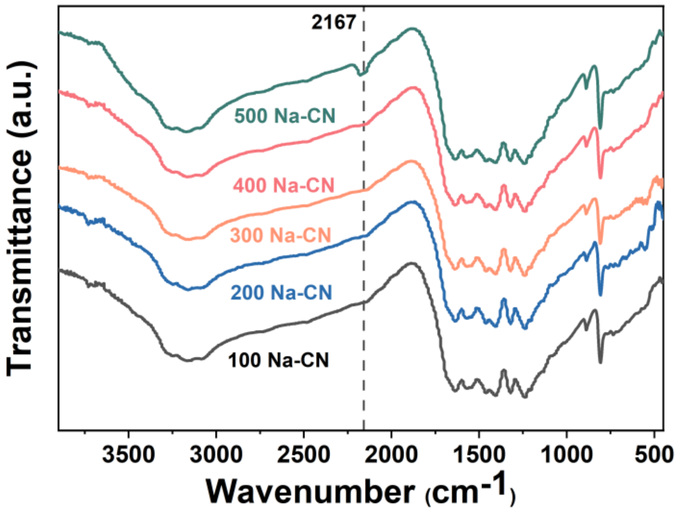

Figure S3. FTIR spectra of y Na-CN.

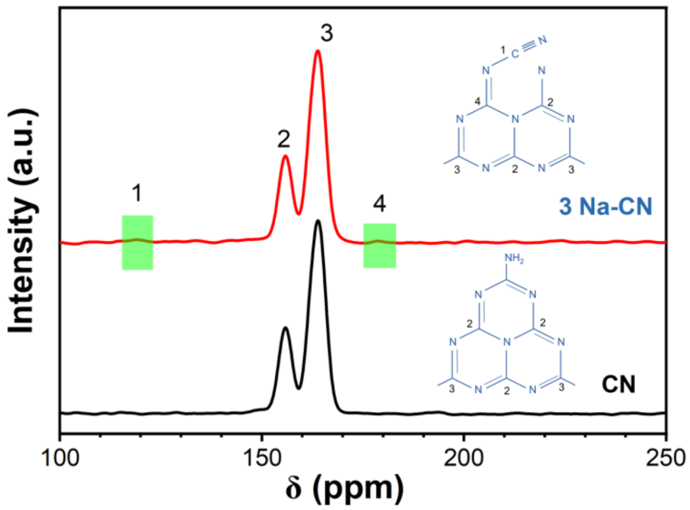

Figure S4. ^{13}C NMR of CN and 3 Na-CN.

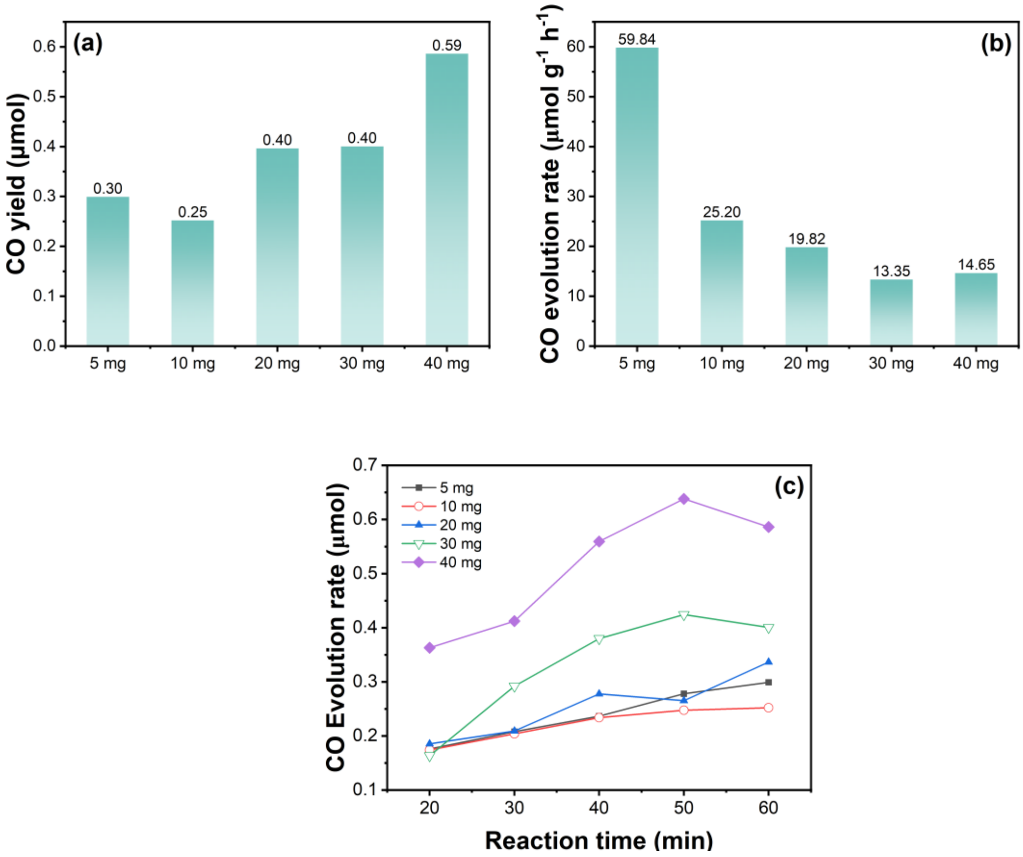

Figure S5. Photocatalytic CO_2 reduction with different catalyst dosages for (a) total CO production, (b) CO unit yield, and (c) kinetic profile.

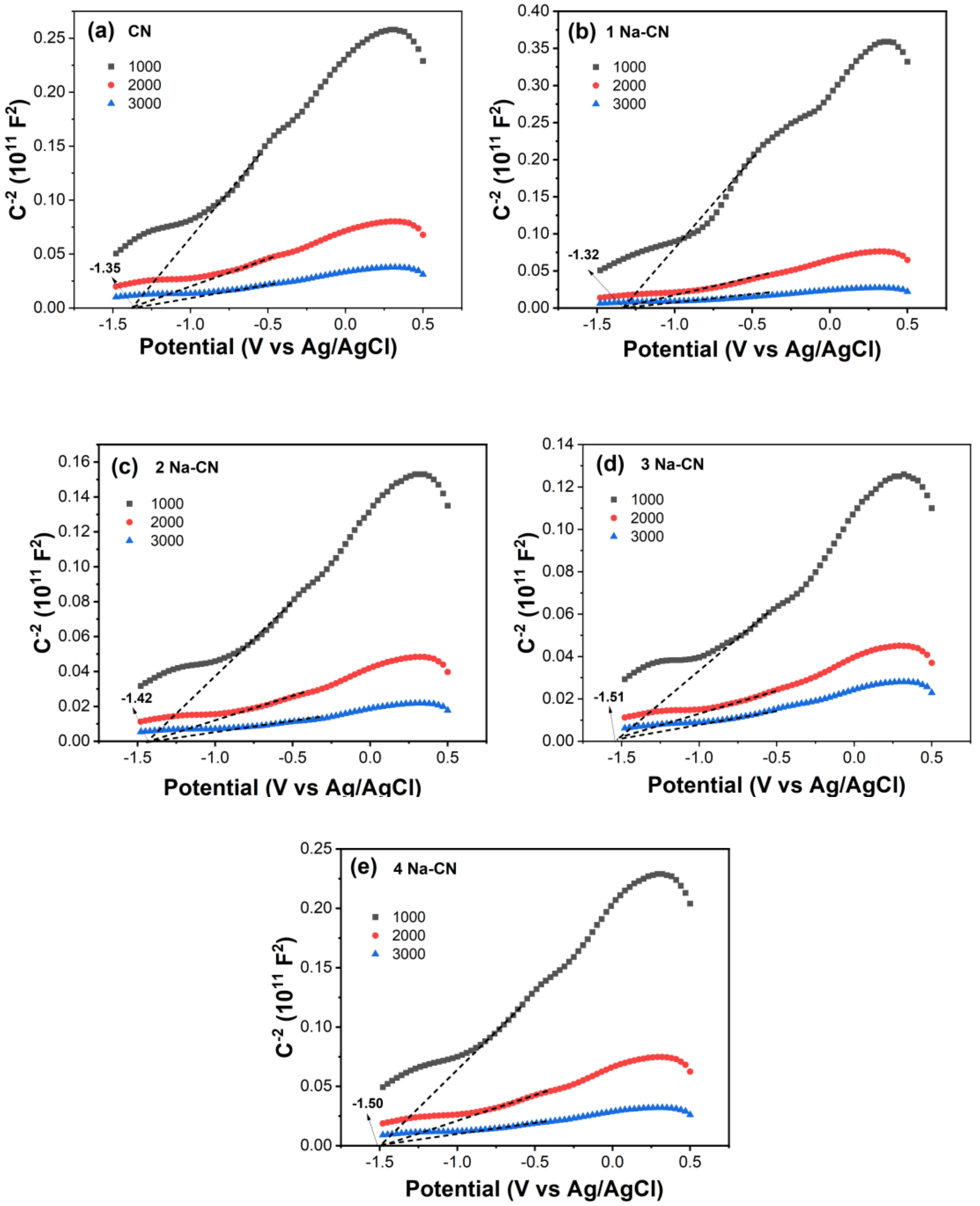

Figure S6. Mott-Schottky plots of (a) CN, (b) 1 Na-CN, (c) 2 Na-CN, (d) 3 Na-CN and (e) 4Na -CN.

References

1. Hou, Y.; Guan, H.; Yu, J.; Cao, S. Potassium/oxygen co-doped polymeric carbon nitride for enhanced photocatalytic CO_2 reduction. *Appl. Surf. Sci.* **2021**, *563*, 150310, doi:10.1016/j.apsusc.2021.150310.
2. Liu, B.; Liu, M.; Tian, L.; Guo, F.; Xia, Y.; Wang, T.; Hu, W.; Guan, R. A novel route to porous N-doping carbon grafted carbon nitride for enhanced photocatalytic activity on CO_2 reduction. *Appl. Surf. Sci.* **2021**, *540*, 148411, doi:10.1016/j.apsusc.2020.148411.
3. Wang, J.; Song, Y.; Zuo, C.; Li, R.; Zhou, Y.; Zhang, Y.; Wu, B. Few-layer porous carbon nitride anchoring Co and Ni with charge transfer mechanism for photocatalytic CO_2 reduction. *J. Colloid Interface Sci.* **2022**, *625*, 722-733, doi:10.1016/j.jcis.2022.04.153.
4. Chen, L.; Yu, J.; Lyu, Z.; Wen, X.; Wang, Y.; Cao, S.; Wang, W. Synergizing interstitial K and substitutional O by dual-doping on carbon nitride for efficient CO_2 photoreduction. *Appl. Surf. Sci.* **2024**, *642*, 158550, doi:10.1016/j.apsusc.2023.158550.
5. Hussien, M.K.; Sabbah, A.; Qorbani, M.; Putikam, R.; Kholimatussadiah, S.; Tzou, D.-L.M.; Elsayed, M.H.; Lu, Y.-J.; Wang, Y.-Y.; Lee, X.-H.; et al. Constructing B─N─P Bonds in Ultrathin Holey g-C_3N_4 for Regulating the Local Chemical Environment in Photocatalytic CO_2 Reduction to CO. *Small* **2024**, *20*, 2400724, doi:10.1002/smll.202400724.

6. Shi, X.; Zhang, Q.; Zhou, Y.; Ye, Q.; Jiang, D.; Tian, D.; Li, D. Boosting charge transfer in Au-decorated B/K co-doped CN nanosheets towards enhanced photocatalytic CO_2 reduction. *Mater. Chem. Front.* **2023**, *7*, 2049-2058, doi:10.1039/D3QM00056G.

7. Liang, Y.; Wu, X.; Liu, X.; Li, C.; Liu, S. Recovering solar fuels from photocatalytic CO_2 reduction over W^{6+}-incorporated crystalline g-C_3N_4 nanorods by synergetic modulation of active centers. *Appl. Catal. B: Environ.* **2022**, *304*, 120978, doi:10.1016/j.apcatb.2021.120978.

8. Yang, Y.; Shen, Z.; Yang, H.; Zou, X.; Meng, Y.; Jiang, L.; Liu, Y.; Xia, Q.; Cao, Y.; Li, X.; et al. Construction adsorption and photocatalytic interfaces between C, O co-doped BN and Pd-Cu alloy nanocrystals for effective conversion of CO_2 to CO. *J. Colloid Interface Sci.* **2023**, *640*, 949-960, doi:10.1016/j.jcis.2023.02.146.

9. Ojha, N.; Bajpai, A.; Kumar, S. Enhanced and selective photocatalytic reduction of CO_2 by H_2O over strategically doped Fe and Cr into porous boron carbon nitride. *Catal. Sci. Technol.* **2020**, *10*, 2663-2680, doi:10.1039/D0CY00343C.

10. Chen, M.; Li, H.; Shen, Z.; Qu, Q.; Yang, W.; Sun, J. A novel sea urchin-shaped copper-incorporated g-C_3N_4 nanoreactor for high-efficiency selectivity photocatalytic CO_2 conversion into CO. *Chem. Eng. J.* **2023**, *476*, 146836, doi:10.1016/j.cej.2023.146836.
